# Think Kidney Function When Testing for and Treating Tuberculosis

**DOI:** 10.34067/KID.0000000578

**Published:** 2024-10-31

**Authors:** Nicola Wearne, Valerie A. Luyckx

**Affiliations:** 1 Division of Nephrology and Hypertension, Groote Schuur Hospital, University of Cape Town, Cape Town, South Africa; 2 Nephrology Department, University Children's Hospital, University of Zurich, Zurich, Switzerland; 3 Department of Public and Global Health, Epidemiology, Biostatistics and Prevention Institute, University of Zurich, Zurich, Switzerland; 4 Renal Division, Brigham and Women's Hospital, Harvard Medical School, Boston, Massachusetts; 5 Department of Paediatrics and Child Health, University of Cape Town, Cape Town, South Africa

**Keywords:** AKI, acute renal failure, AIDS, health equity, diversity, and inclusion, HIV nephropathy, mortality

AKI has been traditionally considered a rarer complication of tuberculosis (TB) and is often attributed to anti tuberculous treatment.^1^ The study in this issue of *Kidney360* by Kansiime *et al.*^2^ challenges this notion, revealing that AKI is more common than previously recognized, occurring in approximately one in three patients within a week of diagnosis of drug-susceptible TB. Notably, AKI occurred before initiation of TB treatment, suggesting that other factors may significantly affect kidney function.

This prospective cohort study enrolled 156 adults diagnosed with drug-susceptible TB across three regional hospitals in Uganda, and aimed to assess the incidence of AKI within the first week of TB diagnosis, identify its predictors, and explore its effect on 30-day survival. TB was diagnosed at an earlier stage in people with HIV (PWH) compared with those without (21 versus 60 days from symptom onset). Earlier diagnosis is likely due to highly structured HIV care in Uganda, which includes routine monitoring and early TB screening. AKI was diagnosed either at day 0, on the basis of an imputed baseline creatinine given that no baselines were available, or at day 7 with a creatinine rise relative to that on day 0. Mortality over 30 days was assessed among those with confirmed AKI on day 7.

The investigators found that 33.3% of participants developed AKI within 7 days of TB diagnosis, with similar rates and stages observed among those with and without HIV. AKI was associated with a markedly high risk of mortality (adjusted hazard ratio, 8.22; 95% confidence interval, 1.94 to 34.72; *P* = 0.0045). This risk underscores the importance of routine kidney function testing at the time of TB diagnosis, before treatment initiation. Contrary to the initial hypothesis, mortality from AKI was lower among patients with TB/HIV coinfection compared with those with TB alone (NS, numbers were small). This counterintuitive finding likely reflects the better overall health status of PWH in this study. The high 30-day mortality (18 patients, 11.5%) was in part affected by the lack of access to dialysis in 15 patients who required it, of whom eight died.

The similar incidence of AKI and noninferior outcomes among those with HIV/TB coinfection compared with TB alone is an important observation and highlights several systemic issues that underlie the global approaches to HIV, TB, and kidney disease. Over the past two decades, much progress has been made in access to diagnosis and treatment of HIV, which resulted from global movements and advocacy. Historically, most of the development aid for health has been allocated to HIV, with a smaller proportion allocated to TB.^3^ Vertical programs have been successful in converting HIV from a universally deadly disease to a chronically controllable disease, but have left other diseases behind. The strong focus on HIV and its complications, as reported by Kansiime *et al.*, led to earlier diagnosis of TB among PWH compared with HIV-negative patients. This highlights the recognition of HIV as a major risk factor of TB, leading to routine TB screening for PWH at diagnosis and symptom onset, along with increased resources for HIV care.^1^ The use of the point-of-care urine lipoarabinomannan assay for diagnosis of active TB in PWH increases the chance of earlier diagnosis.^4^ Urinary lipoarabinomannan is not a reliable tool for TB screening in HIV-negative individuals because of its low sensitivity and high rate of false negatives in this cohort.

The diagnosis of AKI within 1 week of TB diagnosis in this study may reflect the underlying severity of the illness and the possible direct effects of TB. The most common clinical manifestation of TB is pulmonary cavitation, typically associated with productive cough, fever, night sweats, and weight loss. However, *Mycobacterium TB* can spread, particularly to the pleural cavity, lymph nodes, and urogenital tract. Early TB involvement in the kidney may present with proteinuria, pyuria, decreased kidney function, and hematuria, either isolated or accompanied by sterile pyuria.^4^ In a South African biopsy cohort of 316 PWH, in those with granulomatous interstitial nephritis associated with TB, proteinuria was the most common finding (84%), followed by hematuria (60%) and sterile pyuria (35.6%).^5^

A major limitation of this study, acknowledged by Kansiime *et al*.^2^, is the lack of data on the cause and management of AKI. Nephrotoxicity of TB therapy is excluded, but many other possibilities remain, including volume depletion, use of nephrotoxic antipyretics or analgesics, use of traditional remedies, other organ failures, and superimposed infections. Whether these would have been differentially distributed among those with and without HIV is unknown, but it may have been possible that the delay in diagnosis among those without HIV led to a window where there may have been more exposure to nephrotoxins. This may have affected the severity or frequency of AKI in the non-HIV group. In addition, how body mass index, or use of trimethoprim prophylaxis in those on treatment of HIV, may have affected serum creatinine values and diagnosis of AKI is also unknown. Importantly, and not discussed by the authors, is TB-immune reconstitution inflammatory syndrome (TB-IRIS), which is associated with either paradoxical unmasking or worsening of TB after initiation of antiretroviral therapy (ART). Individuals who have reduced CD4 counts and higher HIV RNA viral loads are more susceptible to TB-IRIS. TB-IRIS may directly involve the kidney in the form of TB-granulomatous interstitial nephritis or acute interstitial nephritis.^4^

Globally, TB ranks as the 13th leading cause of death and the second leading infectious disease killer after coronavirus disease 2019, surpassing HIV/AIDS.^6^ This ranking rises to eighth and sixth in low- and lower middle-income countries, respectively.^7^ The combined brunt of both HIV and TB is mainly experienced in low- and lower middle-income countries.^4^ Sub-Saharan Africa is severely and disproportionally affected by the HIV and TB epidemics, with an estimated 19 million people being coinfected.^8^

The prevalence of kidney disease in HIV/TB coinfected patients in Africa is not well documented, and most studies reflect CKD and not AKI. A cross-sectional study in Cameroon reported an 8% prevalence of an eGFR <60 ml/min per 1.73 m^2^ among 200 HIV/TB coinfected patients at different TB treatment stages.^8^ A Ugandan autopsy study of 30 individuals coinfected with HIV and TB, 56% of whom were on ART, identified renal TB in 16 patients (50%), HIV-associated nephropathy in 19%, and other kidney abnormalities in four patients. Autopsy series from India and Mexico, conducted before the widespread use of ART, reported kidney involvement in PWH and TB with respective frequencies of 59% and 23%.^8^ In turn, people with CKD are more vulnerable to TB.

Taken together, the findings by Kansiime *et al.*^2^ highlight that given the strong focus of development aid for health on infectious diseases and the relative neglect of noncommunicable diseases, as well as the fact that kidney disease has been largely overlooked at the global level, important complications, such as AKI, which is associated with significant risk of death, have not been considered in global TB guidelines.^9^ In settings like Uganda, where TB and HIV coinfection is prevalent (HIV increases the risk of TB by 5- to 20-fold)^4^ and resources are limited, integrating regular kidney function assessments into TB care protocols could provide opportunities to significantly improve patient outcomes. Kansiime *et al.*^2^ found the presence of proteinuria or hematuria at enrollment was significantly associated with risk of AKI (odds ratio, 2.68; 95% confidence interval, 1.09 to 6.70; *P* = 0.033). As suggested by others, these markers may serve as early indicators of kidney dysfunction and are easily detectable on urine dipstick. Therefore, screening at TB diagnosis can be simply implemented in resource-limited settings.

The prognosis for TB is generally excellent with good adherence and drug-susceptible strains. Similarly, HIV/TB coinfection has a favorable outlook with timely initiation of ART and TB treatment. However, when kidney disease is present, management and prognosis become more complex. First-line treatment of drug-susceptible *Mycobacterium TB* includes rifampicin, isoniazid, pyrazinamide, and ethambutol during the intensive phase, followed by rifampicin and isoniazid in the continuation phase. Ethambutol is mostly excreted unchanged in urine and requires dose adjustment in kidney failure to prevent the dose-dependent risk of retrobulbar neuritis. Pyrazinamide is primarily metabolized by the liver. Although metabolites are excreted by the kidneys, dose adjustment is not recommended. In patients with HIV/TB coinfection, ART should commence 2 weeks after starting anti-TB treatment, or up to 4 weeks for TB meningitis, to reduce the risk of TB-IRIS.^10^

In summary, despite certain limitations, including the inability to determine the precise cause of AKI at diagnosis and the absence of baseline kidney function data, the study by Kansiime *et al.*^2^ is, to our knowledge, the largest longitudinal prospective cohort study to date exploring the incidence of AKI and short-term mortality among patients with treatment-naïve TB in a resource-limited setting. The study revealed a higher than expected incidence of AKI among people with newly diagnosed TB, which was a strong predictor of short-term mortality. The findings emphasize the importance of kidney function testing at TB diagnosis and the need for further research to understand the underlying mechanisms of AKI and its implications for TB management.
